# Clinical prognosis of isolated anterior cerebral artery territory infarction: a retrospective study

**DOI:** 10.1186/s12883-021-02194-9

**Published:** 2021-04-21

**Authors:** Hyungjong Park, Young Seok Jeong, Seo Hyeon Lee, Seong Hwa Jang, Doo Hyuk Kwon, Jeong-Ho Hong, Sung-Il Sohn, Joonsang Yoo

**Affiliations:** 1 Department of Neurology, School of Medicine, Keimyung University, Daegu, Korea; 2 Department of Neurology, School of Medicine, Yeungnam University, Daegu, Korea; 3 Department of Neurology, Yonsei University College of Medicine, Yongin Severance Hospital, 363 Dongbaekjukjeon-daero, Giheung-gu, 16995 Yongin, Korea

**Keywords:** Anterior cerebral artery, Infarction, Dwelling status

## Abstract

**Background:**

Isolated anterior cerebral artery territory (ACA) infarction is a rare phenomenon, and is known to have distinctive clinical features. Little is known regarding the clinical prognosis of isolated ACA territory infarction with associated factors, and its impact on dwelling and job status. We investigated the short- and long-term outcomes of anterior cerebral artery (ACA) territory infarction, and the associated factors involved in the development of the distinctive symptoms.

**Methods:**

This retrospective study in a prospective cohort of acute ischaemic stroke patients included consecutively enrolled patients with isolated ACA territory infarction. We investigated the functional status using the modified Rankin scale (mRS) score at discharge, three months’ post-discharge, and one-year post-discharge. We also investigated the occlusion site of the ACA (proximal vs. distal); presence of distinctive symptoms of ACA territory infarction including behaviour changes, indifference, aphasia, and urinary incontinence; and the effect of these symptoms on dwelling and job status one year after discharge.

**Results:**

Between April 2014 and March 2019, 47 patients with isolated ACA territory infarction were included. Twenty-nine patients (61.7 %) had good outcomes (mRS ≤ 2) at discharge; however, the mRS score increased at three months (40; 85.1 %, p < 0.001) and one year (41; 87.2 %) post-discharge. Occlusion of the ACA proximal segment was independently associated with the development of distinctive symptoms (adjusted odds ratio, 17.68; 95 % confidence interval: 2.55–122.56, p < 0.05). Twenty-one (48.8 %) patients with good outcomes at one year experienced a change in dwelling status and job loss; 20 (95.2 %) of them had distinctive ACA territory symptoms with proximal ACA occlusion.

**Conclusions:**

Short- and long-term outcomes of isolated ACA territory infarction were favourable. However, proximal segment occlusion was associated with the development of distinctive symptoms, possibly related to future dwelling and job status.

## Background

Isolated anterior cerebral artery (ACA) territory infarction is a rare phenomenon [1], and accounts for only 0.5–3 % of all cerebral infarctions [2–4]. The relative rarity of isolated ACA territory infarction compared to middle cerebral artery (MCA) territory infarction may be related to the fact that the diameter of the A1 segment of the ACA is only approximately half that of the MCA; furthermore, the presence of the anterior communicating artery possibly plays an important role in emboli clearance [5].

Owing to their rarity, there have been few reports regarding the natural prognosis of isolated ACA territory infarctions [2, 3, 6, 7]. Further, in most previous studies, ACA territory infarction was included in the group of hemispheric infarctions, despite its independent vascular topography [8]. ACA territory infarction also has diverse clinical features that are distinct from those of other cerebral vascular territory infarctions, which are not well defined by conventional functional outcome measures focusing on motor function [3, 4, 9, 10]. Therefore, neither the natural prognosis of isolated ACA territory infarction nor the influence of its distinctive symptoms on patients’ outcomes have been revealed.

Thus, we aimed to retrospectively investigate the following: (1) the natural prognosis of patients with isolated ACA territory infarction in short (3-month) and long (1-year) time frames, (2) the factors associated with the development of distinctive symptoms in isolated ACA territory infarction, and (3) the influence of those symptoms of ACA territory infarction on dwelling and job status.

## Materials and methods

### Study population

This study was a retrospective analysis of prospectively registered consecutive patients in a comprehensive stroke center in Korea. All patients experienced an ischaemic stroke within seven days of symptom onset. They were managed according to a standardised protocol and care pathway, based on current guidelines. The inclusion criteria for this study were as follows: (1) isolated ACA territory infarction without involvement of any other cerebral vascular territory on magnetic resonance imaging (MRI); (2) modified Rankin Scale (mRS) ≤ 2 before the qualifying stroke; and (3) followed up at three months and one year after discharge.

During admission, brain MRI, computed tomography (CT) imaging, and cerebral angiography (magnetic resonance angiography, CT angiography, or digital subtraction angiography) were performed for all patients. Systemic evaluations, including 12-lead electrocardiography, chest radiography, standard blood test, and lipid profile, were performed. For detecting the cardiac embolic source, 24-h Holter or electrocardiographic monitoring was performed at the stroke unit. Echocardiography was performed for evaluating the presence of cardiac structural abnormalities or any evidence of cardiac thrombi. Based on these analyses, the stroke aetiology was retrospectively determined according to the Trial of ORG 10,172 in Acute Stroke Management (TOAST) [11] using neuroradiologists’ reports and the consensus of ≥ 2 stroke specialists in weekly stroke conferences.

### Clinical variables and imaging analysis

Data on demographics and stroke risk factors including hypertension, diabetes, smoking status, hyperlipidaemia, and history of coronary artery disease or stroke were obtained. The severity of stroke was determined using the National Institutes of Health Stroke Scale (NIHSS) score at admission.

**Fig. 1:**
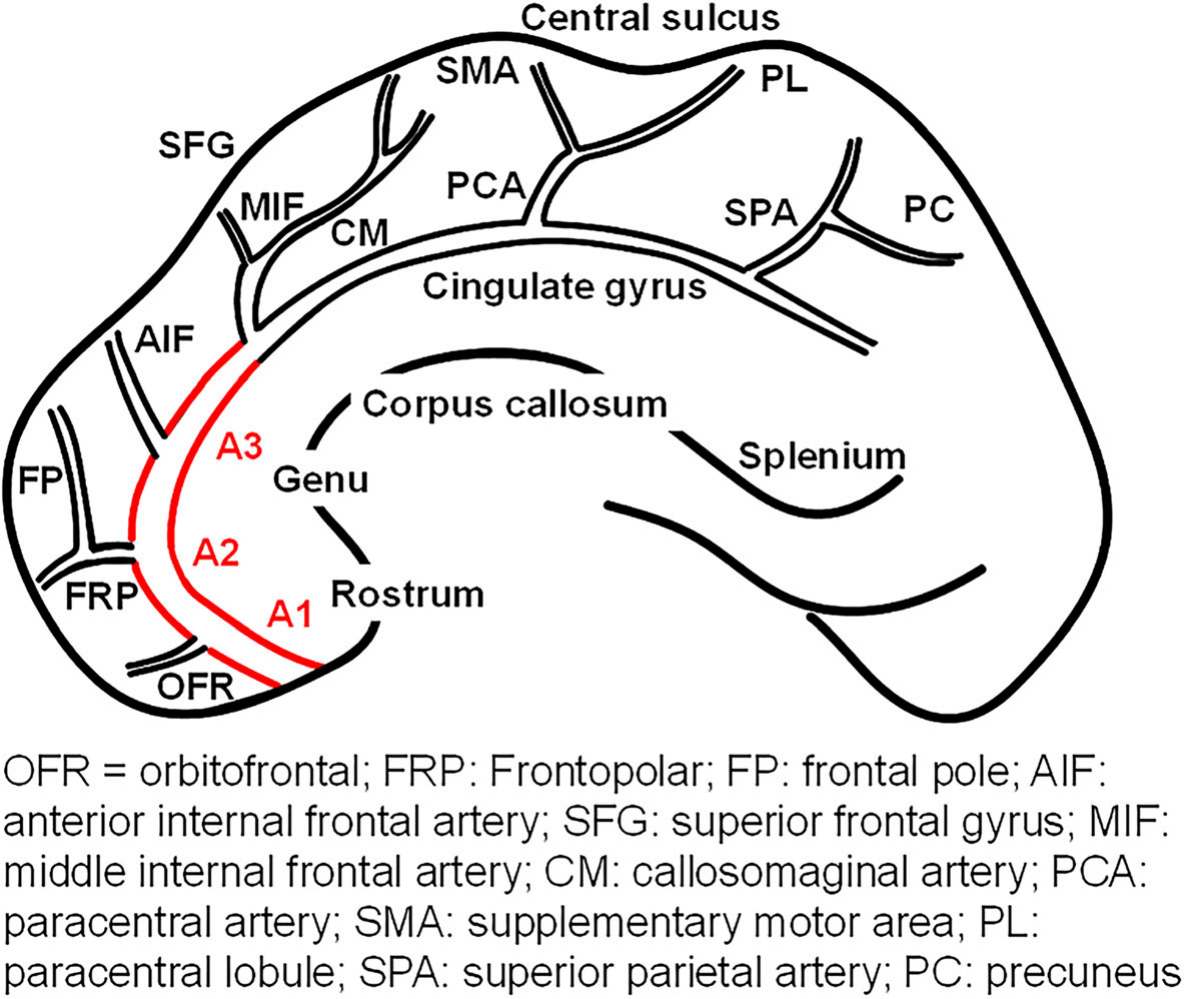
A schematic drawing of anterior cerebral artery (ACA) territory and branches of ACA

Involved vascular territory was determined using high signal intensities on diffusion-weighted images from brain MRIs taken during admission. The location of occlusion or stenosis was also determined by CT angiography or MR angiography. ACA territory was determined according to previous studies [5, 12] (shown in Fig. 1). If cerebral infarction was only involved in the ACA territory without involving any other cerebral vascular territories, the case fulfilled the enrolment criteria. The ACA territory was defined as follows: (1) rostrum, genu, and splenium of the corpus callosum, cingulate gyrus, and frontal pole supplied by the frontopolar and orbitofrontal arteries; (2) medial aspect of the superior frontal gyrus supplied by the anterior and middle internal frontal artery; (3) supplementary motor area supplied by the posterior internal frontal artery; and (4) paracentral lobule and cuneus supplied by the paracentral artery and superior parietal artery. To determine the differences depending on the location more clearly, we divided the ACA region into proximal and distal portions. The proximal portion covered the A1 (precommunicating) to A3 segments (around the genu of the corpus callosum genu), shown as a red line (shown in Fig. 1), and the distal portion covered A4 (terminal branch of A4) and its distal part. The ACA segment was defined according to the Fischer’s classification.

### Outcome measurement

We obtained data regarding the mRS at discharge, three months after discharge, and one year after discharge. The mRS at three months and one year were performed at the outpatient clinic. If the patient missed a scheduled visit, we obtained the information from them or their guardians via telephonic interviews based on a structured questionnaire regarding mRS. Good outcome was defined as mRS 0–2, which is considered to be functionally independent, at three months and one year after discharge. In ACA territory infarction, the cerebral infarction is often not severe; thus, we also investigated the functional status of patients with mRS 0–1. In addition, we also checked other distinctive symptoms related to ACA territory infarction, that could not be measured using mRS [3, 4, 10]. These symptoms included altered consciousness; features of indifference including abulia, akinetic mutism, and apathy; aphasia; behavioral changes such as utilisation, perseveration, and social inhibition; and urinary incontinence [3, 4]. We also investigated the dwelling and job status at three months and one year after discharge.

### Statistical analyses

Data are presented as the mean ± standard deviation, median (interquartile range), or a number (percentage), as appropriate. Differences according to the presence of distinctive symptoms were compared using the chi-square, Fisher’s exact, Student’s t-, and Mann-Whitney U tests, as appropriate. Temporal changes in mRS at discharge, three months after discharge, and one year after discharge were assessed using the chi-square test for trends. To determine the independently associated factors for the development of distinctive symptoms in isolated ACA territory infarction, binary logistic regression analysis was performed, and the results are summarised as odds ratios and 95 % confidence intervals. For multivariable analysis, variables with *p* < 0.1 on univariate analyses were included in the multivariable binary logistic regression analysis. All tests were two-sided, and *p* < 0.05 was considered statistically significant. The final model was fitted using the Hosmer–Lemeshow goodness-of-fit analysis and *p* > 0.05 was considered as the model fit to the binary logistic regression analysis. All statistical analyses were performed using R software version 3.6.1 (R foundation for Statistical Computing, Vienna, Austria).

## Results

### Patients’ characteristics

**Fig. 2:**
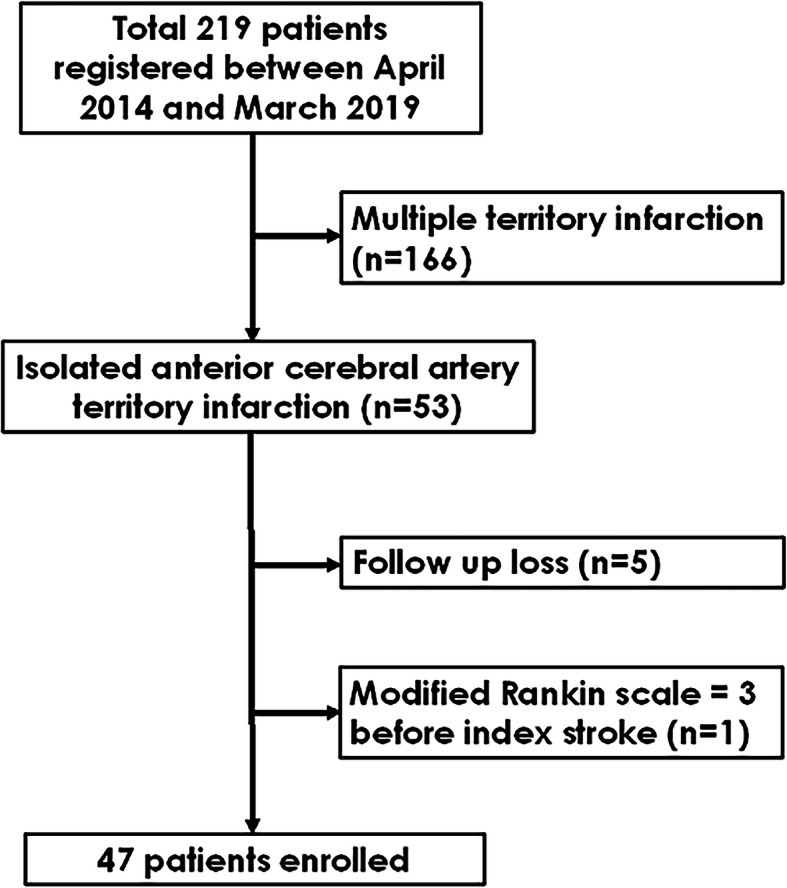
Flow chart of patients’ selection

Between April 2014 and March 2019, a total of 219 patients with cerebral infarction in the ACA territory were admitted in our hospital. Among them, 166 had infarctions in multiple cerebral artery territories. After excluding those patients, 53 with isolated ACA infarction remained. Among these, five patients who were lost to follow-up and one with mRS 3 before onset of stroke were excluded. Finally, 47 patients who fulfilled the inclusion criteria were included in this study (shown in Fig. 2). Among included patients, none underwent endovascular thrombectomy or intravenous tissue plasminogen activator administration. In the acute stage, 35 (74.5 %) and 14 (29.8 %) patients were treated with anti-platelet drugs and anti-coagulants, respectively. Brain protective drugs such as donepezil or choline alfoscerate were used in 13 (27.7 %) patients in the acute stage. In the chronic stage (3 months after stroke onset), new onset atrial fibrillation was detected in 1 patient, and anti-platelet drugs were changed to anticoagulants. The mean age of the included patients was 71.7 ± 11.6 years, and 18 (38.3 %) were male; the initial NIHSS score was 2 (1–5). The most common symptom was weakness of the extremities (33 patients, 70.2 %). The median NIHSS sub-score for motor function in the leg was 2.5, while the median NIHSS sub-score for motor function in the arm was 1. All patients underwent continuous electrocardiographic monitoring, and 39 patients (90.7 %) underwent echocardiography. The most common stroke aetiology was large artery atherosclerosis (*n* = 17, 36.1 %). Patients with distinctive symptoms of ACA infarction had higher initial NIHSS scores and more occlusion of the proximal ACA segment (Table 1).

**Table 1:** Demographic features of patients with ACA territory infarction according to the presence of distinctive symptoms

	Symptom (-) (*N* = 22)		Symptom (+) (*N* = 25)	
**Demographics**				
Age, years	69.1 ± 11.9		73.9 ± 11.2	0.165
Sex, men	9 (40.9)		9 (36.0)	0.964
Initial NIHSS score	1 [0.75–3.25]		4 [1-7]	0.013
**Risk factors**				
Hypertension	10 (45.5)		15 (60.0)	0.481
Diabetes mellitus	5 (22.7)		8 (32.0)	0.702
Hyperlipidemia	4 (18.2)		2 (8.0)	0.545
Atrial fibrillation	5 (22.7)		5 (20.0)	1.000
Smoking	7 (31.8)		8 (32.0)	1.000
Previous stroke	7 (31.8)		3 (12.0)	0.194
Coronary artery disease	3 (13.6)		7 (28.0)	0.399
**Stroke etiology**				0.579
Large artery atherosclerosis	6 (27.3)		10 (40.0)	
Cardioembolism	5 (22.7)		5 (20.0)	
Stroke of other determined etiology	1 (4.5)		0 (0)	
Small vessel disease	0 (0)		0 (0)	
Stroke of undetermined etiology				
Two or more cause	1 (4.5)		0 (0)	
Negative evaluation	9 (41.0)		10 (40.0)	
**Occlusion site, proximal**	9 (40.9)		23 (92.0)	0.001
**Infarction location**				0.317
Right	13 (59.1)		10 (40.0)	
Left	9 (40.9)		14 (56.0)	
Bilateral	0 (0)		1 (4.0)	

Data are shown as n (%), mean ± standard deviation, or median [interquartile range]. *ACA* anterior cerebral artery; *NIHSS* National Institutes of Health Stroke Scale

### Outcomes at discharge, three months after discharge, and one year after discharge

**Fig. 3:**
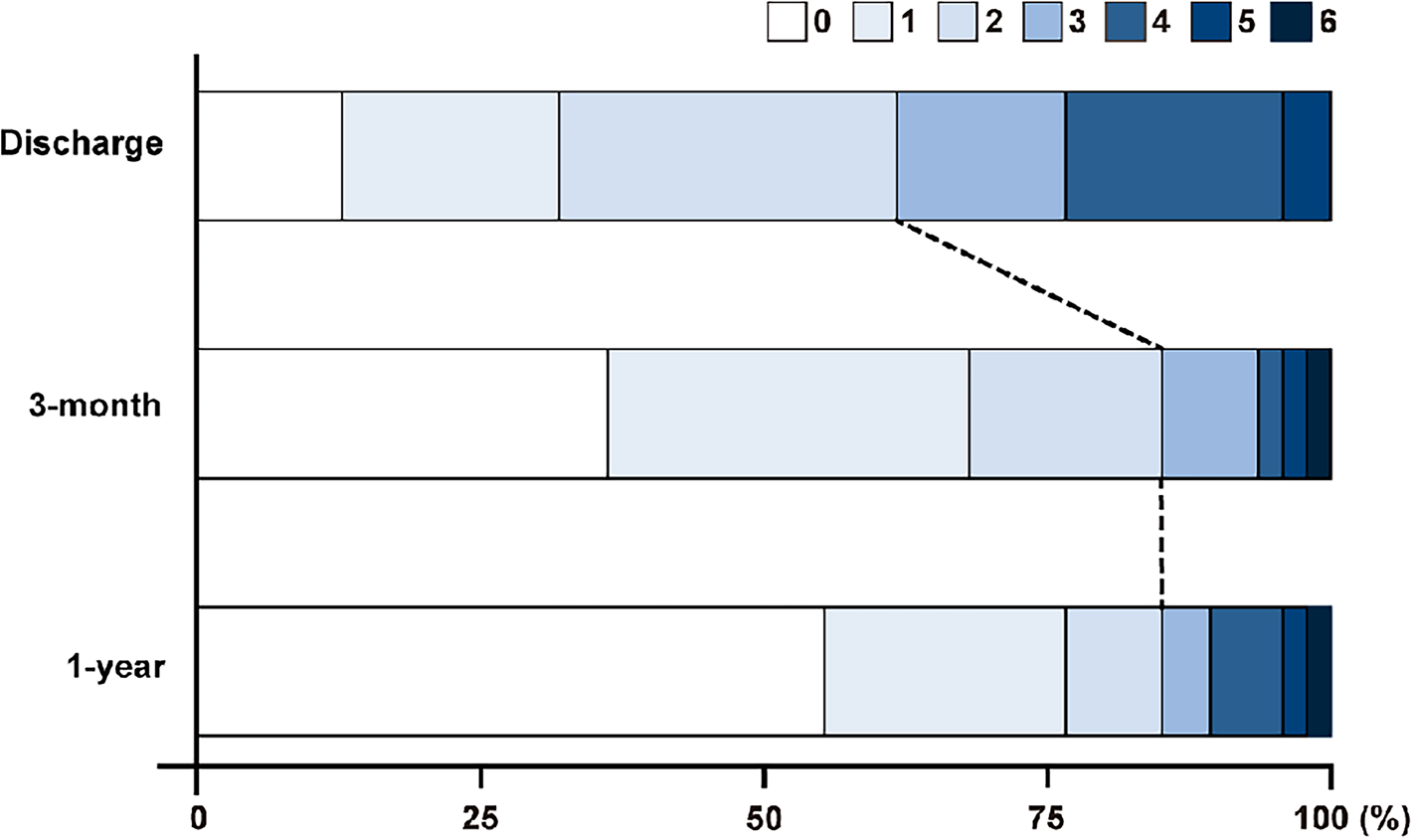
Modified Rankin scale at discharge, 3 months after discharge, and 1 year after discharge

The median mRS at discharge was 2 (1–2); at that point, 29 (61.7 %) patients had good mRS. Short-term (three months) and long-term (one year) mRS after discharge were 1 (0–2) and 0 (0–1), respectively. The number of patients with good mRS increased to 40 (85.1 %) at three months (*p* < 0.001), and one patient (2.1 %) died within three months after discharge. Finally, 41 patients (87.2 %) achieved a good outcome at one year. Compared to mRS 0–1 at three months (68.1 %), the proportion of mRS 0–1 was 76.6 % at one year; there were no additional deaths during this period. The temporal changes in mRS at discharge, three months after discharge (short-term), and one year after discharge (long-term) are shown in Fig. 3.

### Associated factors for the development of distinctive symptoms in ACA territory infarction

Among the 40 patients with a good outcome at 3 months, 25 (62.5 %) had distinctive symptoms with ACA territory infarctions. Among them, 16 had features of indifference and 9 had behavioural changes. In addition, two patients had aphasia, and one suffered from urinary incontinence and voiding difficulty; 23 (92.0 %) had occlusion of the proximal ACA portion. No patient showed improvement of previously distinctive symptoms at three months or one year after discharge. The initial NIHSS score and proximal ACA segment occlusion were significantly associated in cases developing distinctive symptoms with ACA territory infarction. On multivariable analysis, occlusion of the proximal ACA portion (odds ratio, 12.37; 95 % confidence interval, 2.63–92.04; *p* < 0.05) was independently associated with the development of distinctive symptoms in ACA territory infarction (Table 2).

**Table 2:** Factors associated with distinctive symptoms in anterior cerebral artery territory infarction

	Univariable analysis		Multivariable analysis	
	OR (95 % CI)	*P* value	OR (95 % CI)	*P* value
Age	1.04 (0.99–1.10)	0.73		
Sex	0.81 (0.25–2.26)	0.17	2.43 (0.43–13.71)	0.32
Initial NIHSS score	1.26 (1.05–1.65)	< 0.05	1.20 (0.93–1.52)	0.16
Previous stroke	0.44 (0.77–2.55)	0.36		
Coronary artery disease	2.46 (0.59–12.82)	0.238		
Hypertension	1.80 (0.57–5.88)	0.28		
Diabetes mellitus	0.70 (0.19–2.59)	0.32		
Hyperlipidemia	0.39 (0.05–2.24)	0.31		
Atrial fibrillation	0.85 (0.20–3.54)	0.82		
Smoking	1.01 (0.29–3.52)	0.99		
**Occlusion site**				
Distal	Reference		Reference	
Proximal	16.61 (3.68–121.08)	< 0.05	17.68 (2.55–122.56)	< 0.05
**Infarction location**				
Left	Reference			
Right	2.02 (0.63–6.55)	0.24		
Bilateral	NA	NA		
**Stroke etiology**				
Large artery atherosclerosis	Reference			
Cardioembolism	2.50 (0.64–9.66)	0.18		
Stroke of other determined	NA	NA		
Undetermined	0.64 (0.13–3.25)	0.59		

*OR *odds ratio; *CI* confidential interval; *NIHSS* National Institutes of Health Stroke Scale; *NA* Not available

### Dwelling, job status, and distinctive symptoms in ACA territory infarction

Prior to the qualifying stroke, all patients enrolled in this study dwelled in their home with their family and 12 (25.5 %) had a job. Among the 41 (87.2 %) patients classified as having good mRS at 1 year, 17 (36.2 %) moved into chronic care facilities and 4 (33.3 %) lost their jobs. Among the 21 patients who moved their residence or lost their jobs, 20 (95.2 %) had distinctive symptoms of ACA territory infarction, and all of them had proximal ACA segment occlusion.

## Discussion/Conclusion

This study showed that (1) short- and long-term prognoses in isolated ACA territory infarction were good, (2) proximal ACA segment occlusion was an independently associated factor for developing distinctive symptoms in ACA territory infarction, and (3) the distinctive symptoms related to proximal ACA segment occlusion influenced dwelling and job status, regardless of positive mRS.

Short-term mortality after isolated ACA territory infarction can range between 0 and 8 %, which is much lower than the 17.3 % short-term mortality after MCA territory infarction [3, 8, 13]. Consistent with previous studies, only 1 patient (2.1 %) died during the follow-up period in our study. Regarding functional outcomes, approximately 70 % of patients achieved functional independence at 3 months in previous studies [13, 14]. In our study, 85.1 % of patients had a good outcome at 3 months with isolated ACA territory infarction. Thus, short-term outcomes after isolated ACA territory infarction seemed to be favourable, and were line with previous research. However, little is known regarding the long-term prognosis after isolated ACA territory infarction. We investigated the long-term outcomes, with 87.2 % of patients showing good outcomes at one year after discharge. Thus, similar to the short-term outcomes, the long-term outcomes in isolated ACA territory infarction also seem to be favourable.

The most common symptom in isolated ACA territory infarction was motor deficit, typically involving the lower extremity contralateral to the infarction site [3, 4, 8]. As suggested in a previous report, pure motor stroke is the most frequent lacunar syndrome; it is known to involve an upper motor neuron lesion secondary to a lacunar infarct in the ACA or MCA territory [15]. In our study, the median NIHSS sub-score for the leg was also higher than that for the arm [3]. Similar to previous studies, the symptoms in our patients also involved the lower extremity more than the upper extremity. This dominance of the involvement of the lower extremity originates in the paracentral lobule located in the ACA territory. On the contrary, the cortical area for the hand and arm is located in the MCA territory; the corona radiata, where the projection fibres from the cortex merge, is also located in the MCA territory. Therefore, the development of hemiparesis is relatively uncommon in isolated ACA territory infarction, and preserving motor function in the arm may help achieve functional independence. In addition, motor function of the lower extremity usually recovers faster and more completely than that of the upper extremity [16]. This may be the reason why patients with isolated ACA territory infarction showed favourable functional independence as measured by mRS.

Distinctive symptoms in ACA territory infarction include altered mental status, abulia, mutism, decreased verbal fluency, aphasia, and urinary incontinence [3, 4]. Abulia and mutism are associated with cingulate gyrus and supplementary motor area involvement; these areas are important for human behaviour [17–19]. Aphasia is associated with involvement of the supplementary motor area located in the superior medial frontal lobe [20, 21]. Urinary incontinence suggests involvement of the superior frontal gyrus, cingulate, and large infarction lesions affecting the superior and medial parts of the frontal lobe [22]. In terms of distinctive symptom development in ACA territory infarction, infarct size may be an influencing factor. Moreover, the structures associated with distinctive symptoms in the ACA territory are located at least above the A3 segment, and were classified as the proximal portion in our study. Thus, the risk of developing distinctive symptoms in the ACA territory seems to be high in cases of proximal ACA segment occlusion. As mentioned above, proximal ACA segment occlusion was an independent factor for the development of distinctive symptoms in ACA territory infarction.

Furthermore, the presence of distinctive symptoms in ACA territory infarction was closely associated with dwelling and job status in patients with good mRS. According to the mRS, patients with a slight disability who were able to look after their own affairs without assistance, but unable to perform all previous activities, were assigned a score of 2. However, if patients had mild aphasia or abulia with a mild degree of motor deficit, they could still take care of their own affairs as motor function was relatively good. In such cases, their outcome may have been classified as good, even if the patient’s family suffered from the after-effects of the distinctive symptoms of ACA territory infarction. Thus, the real prognosis after ACA territory infarction may not be correctly assessed by mRS alone; it may be wrong to conclude, based on mRS, that patients with isolated ACA territory infarction had favourable outcomes.

Currently, mechanical thrombectomy (MT) is recommended as primary treatment for MCA and carotid artery occlusion [23, 24]. Owing to its rarity, there are few studies on MT in ACA occlusion; the average A2 and A3 diameters were > 2.0 mm [25]. Thus, considering the minimal stent retriever diameter (3.00 mm), the proximal ACA segment was suitable for MT [26]. In a retrospective study in 30 patients, MT on ACA occlusions led to a good recanalization rate with few complications [27]. In addition, recent studies have shown that the presence of ACA occlusion by secondary embolism during MT for MCA without recanalization was associated with poor functional outcomes [28, 29]. Thus, if patients with proximal ACA segment occlusion arrived within the right therapeutic window of time, MT could be considered because proximal segment occlusion was associated with the development of distinctive symptoms in ACA territory infarction; this could affect future patient’s true prognosis, which could not be assessed with mRS alone.

This study had several limitations. First, this was a retrospective single-center study with a small sample size. We did not use a validated questionnaire or examination to check for distinctive symptoms in ACA territory infarction. To circumvent these limitations, we plan to perform a prospective study with a validated questionnaire for prognosis in ACA territory infarction.

Both short- and long-term prognoses based on mRS in isolated ACA territory infarction were favourable. However, despite good mRS, proximal ACA segment occlusion was associated with the development of distinctive symptoms in ACA territory infarction; this could affect the patients’ dwelling and job status. Acute treatment such as endovascular thrombectomy or administration of intravenous tissue plasminogen activator may be beneficial to patients with proximal occlusion.

## Data Availability

The datasets generated and/or analysed during the current study are not publicly available due them containing information that could compromise research participant privacy/consent but are available from the corresponding author on reasonable request.

## Abbreviations

ACA: anterior cerebral artery
CT: computed tomography
MCA: middle cerebral artery
MRI: magnetic resonance imaging
MT: mechanical thrombectomy
NIHSS: National Institutes of Health Stroke Scale
TOAST: Trial of ORG 10172 in Acute Stroke Management
